# Peripheral Spondyloarthritis Presenting with Fever and Severe Systemic Inflammatory Response Mimicking Infection: A Case Series and Literature Review

**DOI:** 10.1155/2023/6651961

**Published:** 2023-07-19

**Authors:** Ibrahim Abdulmomen, Eman Satti, Basem Awadh

**Affiliations:** Rheumatology Division, Department of Medicine, Hamad General Hospital, Doha, Qatar

## Abstract

**Objective:**

To describe four peripheral spondyloarthritis patients presenting with fever and severe systemic inflammatory response mimicking infection.

**Methods:**

Between 2017 and 2019, four patients with the final diagnosis of peripheral spondyloarthritis had atypical presentation of fever and severe systemic inflammatory response requiring hospital admission and extensive workup.

**Results:**

We reported four patients who were admitted to the hospital for fever and arthritis. They all had laboratory tests of the severe systemic inflammatory response (leukocytosis, thrombocytosis, high ESR, and high CRP) concerning infection. They underwent extensive workup for infectious causes, including septic arthritis, which came back negative. Other rheumatic diseases that are known to present with fever such as adult-onset Still's disease, reactive arthritis, and crystal arthritis were all excluded. The final diagnosis of spondyloarthritis was made during their follow-up: three patients with peripheral spondyloarthritis and one with psoriatic arthritis. All patients received conventional DMARDs (methotrexate and sulfasalazine) and two patients received tumor necrosis factor inhibitors in addition to conventional DMARDs to control their disease.

**Conclusion:**

We observed a subgroup of peripheral spondyloarthritis patients presenting with fever and severe systemic inflammatory response requiring hospitalization. Recognition of this subgroup is important and should be considered once an infection is ruled out.

## 1. Introduction

Spondyloarthritides (SpA) comprises a heterogeneous group of inflammatory joint diseases, including psoriatic arthritis (PsA), ankylosing spondylitis (AS), reactive arthritis (ReA), arthritis associated with inflammatory bowel disease (IBD), and undifferentiated spondyloarthritis (uSpA) [1]. Later, the Assessment of SpondyloArthritis International Society (ASAS) substituted this phenotypical approach with a more comprehensive classification system for axial (axSpA) and peripheral spondyloarthritis (pSpA) in which the predominant symptomatology determines the classification [2].

Except for reactive arthritis, SpA patients typically follow a chronic course and they rarely present with fever [3].

## 2. Materials and Methods

This case series review took place at Hamad General Hospital between January 2017 and December 2019 in compliance with the Helsinki Declaration. It was approved by the Medical Research Center and Institutional Review Board at Hamad Medical Corporation.

## 3. Results

### 3.1. Case 1

A 39-year-old Bangladeshi male presented with fever (temperature 38.9°C) and acute left knee pain and swelling for 3 days. His initial workup revealed leukocytosis (white blood cell (WBC) count 15.3 × 103*µ*l) and very high inflammatory markers: C reactive protein (CRP) 141 mg\l (normal < 5) and ESR 92 mm/hr. His knee joint synovial fluid cell count was 65,000*µ*l, and no crystals were seen.

He was treated initially as septic arthritis with antibiotics and washout (blood and synvail fluid cultures remain negative). Thirteen days after his left knee swelling, he developed right knee swelling. Workup for inflammatory arthritis revealed positive HLAB-27. Screening sacroiliac joint X-rays showed a left sacroiliac joint with small localized possible erosions, and the right sacroiliac joint was unremarkable. An MRI of his sacroiliac joints showed bilateral sacroiliitis; thus, he was diagnosed with spondyloarthritis “mixed pattern with predominant peripheral involvement.” He was started on methotrexate with a good response of his peripheral disease, and he required naproxen as needed for his back pain which was infrequent and very mild.

### 3.2. Case 2

A 48-year-old Palestinian male presented with fever (temperature 38.0°C) and one-week history of acute polyarthritis involving the right elbow, right wrist, and left 3rd MCP joints. The patient had a family history of psoriasis affecting his sister. His workup showed leukocytosis (WBC 14.6 × 103*µ*l) and very high inflammatory markers: CRP 275 mg\l and ESR 44 mm/hr. His infection workup was negative. During his hospital admission, he developed right knee swelling and his synovial fluid cell count was only 875/*µ*l and the clinical impression was periarthritis/tendinitis. He was diagnosed with psoriatic arthritis, and sulfasalazine was started with a good response. A few months later, he developed severe bilateral scleritis, so infliximab was added to control his eye disease.

### 3.3. Case 3

A 28-year-old Bangladeshi male presented with two weeks history of fever (temperature 39.0°C) and bilateral knee and left ankle pain and swelling.

Laboratory workup showed leukocytosis (WBC 23 × 103\*µ*l), very high CRP (278 mg\l), and elevated ESR 42 mm/hr. His left knee synovial fluid (SF) WBC count was 12,188/*µ*l. Blood and synovial fluid cultures were negative, and TB QuantiFERON was negative. Later, his HLAB27 came back positive; as he continued to spike a fever, a CT scan of the abdomen was done which showed mild hepatosplenomegaly; bone marrow aspiration revealed no infection or malignancy. After excluding infection as a cause of his fever, a trial of diclofenac potassium 50 mg BID was given and intraarticular 80 mg methylprednisolone was injected into his left knee.

He was diagnosed with peripheral spondyloarthritis and methotrexate, and then adalimumab were started with good control of his disease.

### 3.4. Case 4

A 23-year-old Nepalese male presented with 10 days history of fever (temperature 38.6°C) and acute left knee pain and swelling. Laboratory tests revealed leukocytosis (WBC 15 × 103*µ*l) and very high CRP 195 mg\l. His left knee synovial fluid analysis showed WBC count of 55,375/*µ*l, and no crystals were seen. The patient underwent arthroscopy and washout, and IV antibiotic was started. His blood cultures and synovial fluid cultures were negative. Blood TB gold QuantiFERON and synovial fluid PCR for TB were negative.

Three weeks after discharge, he presented to the clinic with recurrent left knee effusion and he started developing right knee pain and mild swelling.

His inflammatory arthritis workup revealed positive HLAB-27. His sacroiliac joint MRI was normal.

The diagnosis of peripheral spondyloarthropathy was made. He was given an intraarticular steroid injection in his left knee, and sulfasalazine was started. During his subsequent follow-up visits, he had a very good response and no more attacks of arthritis were observed.

## 4. Discussion

In this case series, we report four male patients presenting with fever and very high inflammatory markers, requiring hospital admission and extensive workup. Two patients underwent arthroscopic surgery for presumptive septic arthritis. Their final diagnosis was peripheral spondyloarthritis. Two patients responded to conventional DMARD monotherapy and two required escalation to TNF inhibitors. Most of their laboratory abnormalities returned to normal after two months of treatment (Table 1).

Spondyloarthritis patients can present with a wide range of extra-articular symptoms such as skin rash, diarrhea, and eye discomfort. Fever has been known to be associated with one subtype of SpA, reactive arthritis [4]. In one study, 33% of patients with ReA had a fever when they came to the hospital [5]. However, in the absence of preceding GI/GU infection that is typically seen in reactive arthritis, fever as an extra-articular or systemic manifestation of non-ReA-SpA has not fully been understood. Fever has also been described in inflammatory bowel disease-associated SpA, typically associated with gastrointestinal symptoms [6]. The Italian SpA-IBD expert panel group proposed fever as one of the minor red flags that, along with two other minor criteria, raises the possibility of coexisting IBD [7].

Spondyloarthritis was recently suggested to be a “mixed-pattern disease,” ranked in between autoimmune and autoinflammatory diseases. Autoinflammatory diseases (AInfD) are characterized by strong activation of the innate immune system, they have no detectable autoantibodies, and fever is the most common symptom [8]. Periodic fever syndrome (like familial Mediterranean fever FMF), Gout, and Behçet disease are paradigmatic autoinflammatory diseases. On the other hand, autoimmune diseases (AID) are clinical syndromes caused by the activation of T cells or B cells, or both, in the absence of an ongoing infection or other discernible causes. They are characterized by female predominance, by the presence of serum autoantibodies detected some years before the development of clinical manifestations, and by B or T cell selection defect responses to autoantigens [9]. Passive transfer of these autoimmune factors to susceptible animals can result in the induction of the AID [10]. Rheumatoid arthritis (RA) and systemic lupus erythematosus (SLE) are good examples of AID.

In spondyloarthritis, T cells are key pathogenetic players. Typically (hence called seronegative arthritis), they are not considered to be associated with specific autoantibodies. Although several studies report on the presence of autoantibodies (autoantibodies to carbamylated proteins (anti-CarP) and anti-PsA peptides in psoriatic arthritis), the pathogenicity of these autoantibodies has not been established [11–14]. The lack of a female preponderance, absence of detectable autoantibodies, and lack of response to steroids argues against classification as AID and is in favor of SpA being an AInfD [1, 15–17].

At the molecular level, the human leukocyte antigen (HLA)-B27 allele is present in 85–90% of AS cases [18]. Accumulation of nonconventional forms of HLA-B27, such as free heavy chains, was recently reported in the gut and synovial tissue of SpA patients, as well as in HLA-B27 transgenic rats [19]. Non-HLA genes, involved in innate immune recognition and cytokine signaling pathways, are linked with SpA. Such genes include tumor necrosis factor (TNF) and IL-23, which are shared with IBD and psoriasis [20]. On the other hand, thus far no IL-1 gene cluster mutations have been associated with SpA which is against the definition of SpA as an AInfD [21].

Spondyloarthritis is difficult to diagnose when the patient's main symptom is fever, often resulting in hospitalization for further diagnostic workups. Febrile undiagnosed SpA patients can undergo unnecessary arthroscopic surgery because of suspected septic arthritis. A retrospective study carried out by Byun et al. showed that only 7.7% of febrile SpA patients received their initial subspecialty evaluation with rheumatologists, suggesting that many clinicians do not consider SpA in the evaluation of febrile patients with extra-articular SpA symptoms [3]. They reported twenty-six SpA patients who initially presented with fever, 92.3% were hospitalized and three febrile SpA patients (11.5%) underwent unnecessary arthroscopic surgery. When matched with 100 SpA patients without fever, AS was significantly less common in the febrile SpA group than in the control group. Febrile SpA patients had peripheral SpA more commonly than control SpA patients.

In addition to fever, all our cases had an acute presentation with a severe systemic inflammatory response that requires hospitalization and further workup before the final diagnosis was reached. None of our patients had gastrointestinal or genitourinary symptoms to suggest reactive arthritis or IBD-associated SpA. We believe that this subgroup of SpA patients behave similarly to autoinflammatory diseases (AInfDs) at presentation. Similar to the previous observation by Jin Byun et al., all our cases had a predominant peripheral presentation of SpA.

## 5. Conclusion

Severe systemic inflammatory response, mimicking infection, can be the first and the most predominant presentation in a subgroup of patients with spondyloarthritis. In addition to infectious causes and other rheumatic diseases that are known to present with fever and arthritis, spondyloarthritis should be considered in the differential diagnosis.

## Figures and Tables

**Table 1:** Summary of patients' presentation, laboratory results, and treatment.

Cases	Sex	Age (y)	Nationality	Fever	Presentation	Synovial fluid cell count (*µ*l)	Laboratory results	HLA B27	Response to DMARDs/NSAIDs
1	Male	39	Bangladeshi	Yes	Three days of acute left knee arthritis	65,000	CRP = 141	Present	Methotrexate/naproxen prn
							ESR = 92		
							WBC = 15.3		
							Plat = 418		

2	Male	48	Palestinian	Yes	One week of acute oligoarthritis	875	CRP = 275	Absent	SSZ + infliximab
							ESR = 44		
							WBC = 14.6		
							Plat = 539		

3	Male	28	Bangladeshi	Yes	Two weeks of acute oligoarthritis	12,188	CRP = 278	Present	Methotrexate adalimumab
							ESR = 42		
							WBC = 23		
							Plat = 486		

4	Male	23	Nepalese	Yes	Ten days of acute left knee arthritis	55,375	CRP = 195	Present	Sulfasalazine
							WBC = 15		
							Plat = 338		

WBC = white blood cell, CRP = C-reactive protein, ESR = erythrocyte sedimentation rate, plat = platelets, prn = pro re nata is a Latin term that means as needed, MTX = methotrexate, and SSZ = sulfasalazin.

## Data Availability

The data presented in this study are available from the corresponding author on reasonable request.

## Additional Points

*Key Message*. A subgroup of peripheral spondyloarthritis patients can present with fever and severe systemic inflammatory response. We hypothesize that these patients behave similar to patients with autoinflammatory diseases.
